# Factors Associated With the Antimicrobial Resistance Profile of Uropathogenic *Escherichia coli* in a Referral Hospital in Northern Peru

**DOI:** 10.1155/ipid/4524106

**Published:** 2026-10-07

**Authors:** Christian Alberto Rodriguez-Saldaña, Daniela Valeria Clavijo-Gallardo, Victor Serna-Alarcón, Luis Gabriel Farfán-Chávez, Guiovanna María Wong-Terrones, Daniel Reyes-Chávez

**Affiliations:** ^1^ Faculty of Human Medicine Antenor Orrego Private University Piura Peru; ^2^ Department of Infectious Diseases Peru-Corea Santa Rosa II-2 Hospital Piura Peru; ^3^ Department of Infectious Diseases José Cayetano Heredia III-1 Hospital EsSalud Piura Peru essalud.gob.pe; ^4^ Department of Internal Medicine José Cayetano Heredia III-1 Hospital EsSalud Piura Peru essalud.gob.pe

**Keywords:** antimicrobial resistance, *Escherichia coli*, urinary tract infections

## Abstract

**Introduction:**

Antimicrobial resistance in uropathogenic *Escherichia coli* (UPEC) compromises urinary tract infection management, yet analytical evidence from northern Peru remains limited. This study evaluated demographic and clinical factors associated with resistance in UPEC isolates from a regional referral hospital.

**Methods:**

A retrospective analytical cross‐sectional study included 350 patients with monomicrobial urine cultures positive for UPEC during 2024. Resistance was defined as resistance to at least one of 19 antimicrobials. Associations were estimated using modified Poisson regression with robust variance.

**Results:**

Overall, 323 isolates (92.3%) showed resistance. The highest resistance proportions were observed for ciprofloxacin (70.9%), ceftriaxone (65.4%), ceftazidime (62.0%), and trimethoprim/sulfamethoxazole (46.3%). Age showed a significant nonlinear association with resistance (*p* = 0.033). No statistically conclusive independent associations were observed for sex, prior antibiotic use, diabetes mellitus, hypertension, or obesity after adjustment.

**Conclusion:**

UPEC resistance was highly frequent and concentrated in commonly used agents, supporting local surveillance and susceptibility‐guided empirical therapy.

## 1. Introduction

Urinary tract infections (UTIs) are among the most common bacterial infections encountered in outpatient, emergency, and hospital care. Their clinical spectrum ranges from lower UTIs, such as cystitis, to pyelonephritis and, in severe cases, bacteremia or sepsis. Their management has become increasingly difficult because antimicrobial resistance compromises the effectiveness of standard therapies and contributes to prolonged illness, healthcare use, and mortality. In 2019, bacterial antimicrobial resistance was associated with millions of deaths worldwide, establishing it as a major contemporary public health threat [1, 2]. Resistant UTIs also represent a substantial component of this global burden, particularly when caused by *Escherichia coli* resistant to fluoroquinolones or third‐generation cephalosporins [3].

Uropathogenic *Escherichia coli* (UPEC) is the leading cause of uncomplicated UTIs and accounts for a considerable proportion of complicated infections. *E. coli* is a Gram‐negative bacillus belonging to the order Enterobacterales and is normally present in the intestinal microbiota. However, extraintestinal lineages possess characteristics that allow them to colonize the periurethral region, ascend through the urinary tract, invade the urothelium, and persist within host tissues [4, 5].

The pathogenicity of UPEC depends on a heterogeneous repertoire of adhesins, type 1 and P fimbriae, toxins, iron‐acquisition systems, capsules, flagella, outer‐membrane proteins, and biofilm‐related mechanisms. These factors promote attachment to urinary epithelial cells, invasion, nutrient acquisition, evasion of host defenses, and intracellular persistence [6, 7]. Colonization of intestinal and urogenital reservoirs may facilitate recurrent migration into the urinary tract, while differences in host microbiota and bacterial lineage can influence persistence and clinical expression [8].

Increasing resistance has reduced the reliability of several antimicrobials traditionally used for empirical treatment, including fluoroquinolones, trimethoprim/sulfamethoxazole, and third‐generation cephalosporins. Resistance may arise through enzymatic drug inactivation, modification of antimicrobial targets, reduced outer‐membrane permeability, active efflux, metabolic adaptation, biofilm formation, and horizontal transfer of resistance determinants [9, 10]. In Latin America, extended‐spectrum β‐lactamases, particularly CTX‐M enzymes, and plasmid‐mediated quinolone resistance are frequently reported and may coexist within the same isolate, producing resistance to several antimicrobial classes [11].

The epidemiology of resistant *E. coli* is not restricted to clinical antimicrobial exposure. Resistant strains and mobile genetic elements may circulate among humans, animals, food products, wastewater, soil, and other environmental reservoirs [12, 13]. This interconnected transmission is especially relevant in Latin American settings where sanitation, antimicrobial access, agricultural practices, and environmental control remain heterogeneous [14]. At the patient level, prior antibiotic exposure, recurrent UTI, diabetes mellitus, healthcare contact, and international travel have been identified as possible determinants of resistance although the consistency and temporal direction of these associations remain uncertain [15].

Because resistance patterns vary according to local prescribing practices, hospital complexity, patient characteristics, circulating bacterial lineages, and laboratory methods, institutional surveillance remains essential. Regional hospital data confirm the predominance of *E. coli* among urinary isolates, but analytical evidence from northern Peru remains limited [16]. This study, therefore, aimed to evaluate demographic and clinical factors associated with antimicrobial resistance in UPEC isolates from patients treated at a regional referral hospital in Piura, Peru. Drug‐specific susceptibility and the distribution of resistance profiles were also described.

## 2. Materials and Methods

### 2.1. Study Design and Setting

We conducted a retrospective analytical cross‐sectional study at José Cayetano Heredia III‐1 Hospital, an EsSalud regional referral hospital located in Piura, northern Peru. The study period extended from January 1 to December 31, 2024. This period was selected because 2024 was the most recent complete calendar year for which the institutional microbiology database and electronic medical records were available with adequate completeness, consistency, and uniformity at the time of data access. The inclusion of an entire calendar year also reduced the potential influence of selecting a particular season or shorter interval.

The study population comprised patients evaluated in outpatient clinics, the emergency department, or inpatient wards who had a clinically diagnosed UTI and a monomicrobial urine culture positive for *E. coli*. For this study, urinary *E. coli* isolates obtained from patients with a confirmed UTI were operationally considered UPEC. Clinical data were obtained from electronic medical records, and microbiological results were retrieved from the institutional laboratory database.

### 2.2. Sample Size

The minimum sample size was calculated using the formula for estimating a single proportion in a population of unknown size as follows:

(1) n=Z12−α/2p1−pd2,

where *n* is the required sample size, *Z*_1−*α*/2_ is the standard normal value corresponding to a 95% confidence level, *p* is the expected prevalence of antimicrobial resistance, and *d* is the maximum accepted margin of error.

An expected prevalence of 70.4% was used based on a previous Peruvian study that reported ciprofloxacin‐resistant *E. coli* among patients with community‐acquired upper UTIs [17]. With *Z* = 1.96, *p* = 0.704, 1−*p* = 0.296, and *d* = 0.05, the estimated sample size was 320.2 records. This value was rounded up to a minimum of 321 patients. The final analysis included 350 eligible records. The calculation was verified using EPIDAT.

### 2.3. Sampling and Unit of Analysis

Participants were selected by simple random sampling from the complete list of eligible urine cultures recorded during the study period. Each eligible record was assigned a unique numerical identifier, and the study sample was selected using a computer‐generated sequence of random numbers.

The unit of analysis was the patient record linked to the corresponding urinary *E. coli* isolate. Only one eligible isolate per patient was included to avoid repeated observations from the same individual.

### 2.4. Eligibility Criteria

Patients of any age with a clinically diagnosed UTI and a monomicrobial urine culture positive for *E. coli* between January 1 and December 31, 2024, were eligible.

Records were excluded when the urine culture was polymicrobial, when the clinical or microbiological information required for the analysis was incomplete, or when a documented terminal‐stage condition could substantially limit the interpretation of the clinical variables evaluated.

### 2.5. Variables and Data Collection

The primary outcome was documented antimicrobial resistance, defined as resistance to at least one of the 19 antimicrobial agents evaluated in the institutional microbiology database. Isolates were classified as having resistance when at least one result was reported as resistant. Results classified as intermediate were retained as a separate susceptibility category and were not included as resistant in the primary binary outcome. This broad endpoint was selected to capture the overall burden of any documented loss of in vitro activity across the complete routine panel and to provide a uniform binary outcome for the association analysis. More restrictive phenotypes, such as multidrug resistance or extended‐spectrum beta‐lactamase production, and resistance to specific empirical agents represent distinct clinical constructs; drug‐specific susceptibility and combined resistance profiles were, therefore, reported separately to preserve clinically relevant detail.

The independent variables were age, sex, prior antibiotic use, diabetes mellitus, arterial hypertension, and obesity. Hospital service was recorded as outpatient clinic, emergency department, or inpatient ward. Prior antibiotic use referred to antibiotic exposure documented in the electronic medical record before the index urine culture and was analyzed as a binary variable. The available records did not provide sufficiently uniform information on the antimicrobial class, dose, duration, indication, or interval between exposure and urine culture.

Data were extracted using a structured collection form that included the patient identifier, demographic characteristics, clinical history, prior antibiotic use, comorbidities, hospital service, and antimicrobial susceptibility results. The comorbidities included in the analysis were restricted to conditions recorded systematically in the institutional database. Other potentially relevant conditions, including immunosuppression, recurrent UTI, urinary catheterization, and structural urinary abnormalities, were not analyzed because their documentation was incomplete or insufficiently standardized.

### 2.6. Antimicrobial Susceptibility Testing

Identification and antimicrobial susceptibility testing were performed by the institutional microbiology laboratory using the VITEK 2 automated system (bioMérieux, Marcy‐l’Étoile, France). This system determines minimum inhibitory concentrations and assigns categorical interpretations using standardized antimicrobial susceptibility criteria. Automated and minimum inhibitory concentration‐based methods are widely used to support reproducible clinical susceptibility testing and institutional surveillance [18, 19].

Minimum inhibitory concentration results were interpreted by the laboratory as susceptible, intermediate, or resistant according to the breakpoints established in the *Clinical and Laboratory Standards Institute M100: Performance Standards for Antimicrobial Susceptibility Testing*, 33rd edition [18]. The research team retained the original laboratory classifications without post hoc reinterpretation. This decision was relevant because the application of different breakpoint systems, particularly CLSI and EUCAST, can modify the categorization of some isolates and affect comparisons between surveillance datasets [20].

The primary resistance outcome was determined using all 19 antimicrobial agents available in the analytical database. Drug‐specific susceptibility distributions were presented for the nine agents included in Figure 1.

**FIGURE 1:**
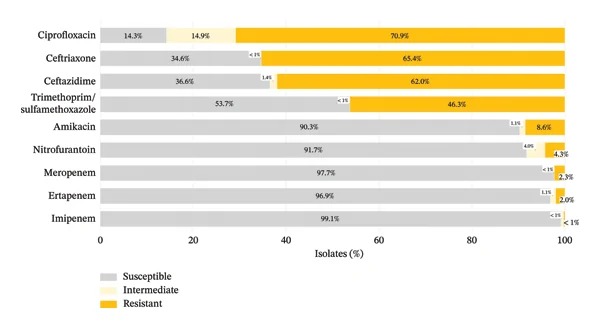
Antimicrobial susceptibility distribution of uropathogenic *Escherichia coli* isolates.

For the resistance‐profile analysis, a summarized panel comprising amikacin, ceftazidime, ciprofloxacin, ceftriaxone, carbapenems, nitrofurantoin, and trimethoprim/sulfamethoxazole was used. Carbapenem resistance was defined as resistance to at least one of ertapenem, imipenem, or meropenem. Profiles involving resistance to four or more panel agents were presented individually. Profiles involving one, two, or three panel agents were grouped according to the number of resistant agents. Isolates with resistance exclusively to antimicrobials outside the summarized panel were classified separately.

### 2.7. Statistical Analysis

Categorical variables were summarized using absolute frequencies and percentages. The distribution of age was assessed using the skewness–kurtosis test. Because age departed from a normal distribution, it was reported as the median and interquartile range.

Age was compared between patients with and without documented resistance using the Mann–Whitney *U* test. Pearson’s chi‐square test was used for bivariate comparisons involving categorical variables. The Fisher–Freeman–Halton exact test was applied to contingency tables with sparse expected frequencies.

Crude prevalence ratios and their 95% confidence intervals were estimated using separate modified Poisson regression models with a log link and robust variance. Adjusted prevalence ratios were estimated using a multivariable modified Poisson model with the same link and variance specification.

Age, sex, prior antibiotic use, diabetes mellitus, arterial hypertension, and obesity were entered simultaneously into the adjusted model. These variables were selected *a priori* according to their clinical relevance and previously reported relationships with antimicrobial resistance. No automated, stepwise, or significance‐based variable‐selection procedure was applied.

The functional form of age was examined by adding a quadratic term to the adjusted model. Because the relationship between age and resistance was nonlinear, age was centered at the sample mean and included in the final model as linear and quadratic terms. The overall contribution of age was evaluated using a joint Wald test.

Multicollinearity was assessed using variance inflation factors obtained from an auxiliary linear model containing the same predictors as the final modified Poisson model. Model specification was evaluated using an auxiliary model that included the linear predictor and its squared term.

Model diagnostics included assessment of convergence, deviance, Pearson chi‐square relative to the residual degrees of freedom, standardized Pearson residuals, and leverage values. Leverage was obtained from an, otherwise, identical Poisson model fitted without robust variance to the same analytical sample. An absolute standardized Pearson residual greater than 3 was considered potentially atypical. Elevated leverage was assessed using the threshold 2_*p*/*n*_, where *p* represented the effective number of estimated parameters and *n* represented the analytical sample size.

A complete‐case analysis was performed because all 350 observations had complete data for the variables included in the multivariable model. All statistical tests were two‐sided, and a *p* value < 0.05 was considered statistically significant.

Statistical analyses were conducted using Stata Version 16.0 (StataCorp LLC, College Station, TX, USA). Figures were generated using R Version 4.5.0 (2025‐04‐11 ucrt; R Foundation for Statistical Computing, Vienna, Austria) and the *ggplot2* package.

### 2.8. Ethical Considerations

The study protocol was approved by the Research Ethics Committee of José Cayetano Heredia III‐1 Hospital, EsSalud Piura (approval code 000016‐CEI‐GR‐RAPI‐ESSALUD‐2025) and by the Institutional Bioethics Committee of Antenor Orrego Private University (Resolution No. 00018‐2025‐UPAO).

The requirement for informed consent was waived because the study involved the retrospective analysis of anonymized clinical and microbiological records without direct contact with patients. All records were coded before analysis, access to the study databases was restricted to the research team, and findings were reported exclusively in aggregate form.

## 3. Results

### 3.1. Patient Characteristics and Antimicrobial Resistance

The analysis included 350 patients, all of whom had complete data for the variables entered into the multivariable model. Resistance to at least one of the 19 antimicrobial agents evaluated was identified in 323 isolates (92.3%); the remaining 27 isolates (7.7%) had no documented resistance. Intermediate susceptibility was retained as a separate category and was not included in the definition of resistance.

The median age was 61 years (IQR, 45–73), and 282 patients (80.6%) were female. Most urine cultures were obtained from outpatient clinics (57.4%), followed by the emergency department (29.1%) and inpatient wards (13.4%). Patients with resistant isolates were older than those without documented resistance (62 [IQR, 48–74] vs 54 [IQR, 36–66] years; *p* = 0.012). Prior antibiotic use was reported in 82.4% of the patients with resistant isolates and in 63.0% of those without resistance (*p* = 0.014). Diabetes mellitus was also more frequent among patients with resistant isolates (67.2% vs 40.7%; *p* = 0.006). Sex, arterial hypertension, obesity, and hospital service did not differ significantly between groups (Table 1).

**TABLE 1:** Demographic and clinical characteristics of patients according to the antimicrobial resistance status of uropathogenic *Escherichia coli* isolates.

Characteristic	Overall (*N* = 350)	No resistance detected (*N* = 27)	Resistance to ≥ 1 antimicrobial (*N* = 323)	*p* value
Age (years)^a^	61 (45–73)	54 (36–66)	62 (48–74)	0.012^b^
Sex, *n* (%)				0.256^c^
Male	68 (19.4)	3 (11.1)	65 (20.1)	
Female	282 (80.6)	24 (88.9)	258 (79.9)	
Prior antibiotic use, *n* (%)				0.014^c^
No	67 (19.1)	10 (37.0)	57 (17.6)	
Yes	283 (80.9)	17 (63.0)	266 (82.4)	
Diabetes mellitus, *n* (%)				0.006^c^
No	122 (34.9)	16 (59.3)	106 (32.8)	
Yes	228 (65.1)	11 (40.7)	217 (67.2)	
Arterial hypertension, *n* (%)				0.410^c^
No	155 (44.3)	14 (51.9)	141 (43.7)	
Yes	195 (55.7)	13 (48.1)	182 (56.3)	
Obesity, *n* (%)				0.406^c^
No	245 (70.0)	17 (63.0)	228 (70.6)	
Yes	105 (30.0)	10 (37.0)	95 (29.4)	
Hospital service, *n* (%)				0.172^d^
Outpatient clinic	201 (57.4)	20 (74.1)	181 (56.0)	
Emergency department	102 (29.1)	4 (14.8)	98 (30.3)	
Inpatient ward	47 (13.4)	3 (11.1)	44 (13.6)	

*Note:* Data are presented as *n* (%) unless, otherwise, specified; percentages were calculated by column. Resistance was defined as resistance to ≥ 1 tested antimicrobial; intermediate results were not classified as resistant. IQR, interquartile range.

^a^Median (interquartile range).

^b^Mann–Whitney *U* test.

^c^Pearson’s chi‐square test.

^d^Fisher–Freeman–Halton exact test.

### 3.2. Antimicrobial Susceptibility

Ciprofloxacin had the highest proportion of resistant isolates (70.9%), followed by ceftriaxone (65.4%), ceftazidime (62.0%), and trimethoprim/sulfamethoxazole (46.3%). Resistance was less frequent for amikacin (8.6%) and nitrofurantoin (4.3%). Among carbapenems, resistance was 2.3% for meropenem, 2.0% for ertapenem, and below 1% for imipenem.

Intermediate susceptibility was most frequent for ciprofloxacin (14.9%) and nitrofurantoin (4.0%); the corresponding proportions for the other agents were low or absent (Figure 1).

Bars represent the percentages of isolates classified as susceptible, intermediate, or resistant to each antimicrobial agent. Values below 1% are displayed as < 1%. Intermediate results were retained as a separate category and were not classified as resistant in the primary binary outcome.

### 3.3. Factors Associated With Antimicrobial Resistance

Under a linear specification, age was associated with resistance in the crude model (cPR, 1.002 per year; 95% CI, 1.000–1.003; *p* = 0.032). Diabetes mellitus was also associated with resistance before adjustment (cPR, 1.095; 95% CI, 1.016–1.181; *p* = 0.017). The crude association for prior antibiotic use did not reach statistical significance (cPR, 1.105; 95% CI, 0.995–1.227; *p* = 0.062). No crude associations were found for female sex, arterial hypertension, or obesity (Table 2).

**TABLE 2:** Crude and adjusted associations with antimicrobial resistance in uropathogenic *Escherichia coli* isolates.

Variable	cPR (95% CI)	*p* value	aPR (95% CI)	*p* value
Age, per 1‐year increase^a^	1.002 (1.000–1.003)	0.032	—^b^	0.033
Female sex	0.957 (0.899–1.019)	0.168	0.975 (0.918–1.037)	0.426
Prior antibiotic use	1.105 (0.995–1.227)	0.062	1.118 (0.942–1.326)	0.201
Diabetes mellitus	1.095 (1.016–1.181)	0.017	1.091 (0.997–1.194)	0.057
Arterial hypertension	1.026 (0.964–1.092)	0.419	0.979 (0.923–1.039)	0.492
Obesity	0.972 (0.906–1.044)	0.436	1.009 (0.930–1.096)	0.821

*Note:* cPRs and aPRs were estimated using modified Poisson regression with a log link and robust variance. The adjusted model included age, sex, prior antibiotic use, diabetes mellitus, arterial hypertension, and obesity.

Abbreviations: aPR, adjusted prevalence ratio; CI, confidence interval; cPR, crude prevalence ratio.

^a^Crude effect per 1‐year increase.

^b^Global Wald test for the linear and quadratic age terms; a single aPR was not reported because the adjusted association was nonlinear.

The relationship between age and resistance was nonlinear. Age was, therefore, centered at the sample mean and entered into the adjusted model as linear and quadratic terms. Their joint contribution was statistically significant (Wald *χ* ^2^[2] = 6.82; *p* = 0.033), precluding interpretation through a single adjusted prevalence ratio per year.

After adjustment, the estimate for prior antibiotic use did not reach statistical significance (aPR, 1.118; 95% CI, 0.942–1.326; *p* = 0.201). The crude association with diabetes mellitus was attenuated and no longer met the significance threshold (aPR, 1.091; 95% CI, 0.997–1.194; *p* = 0.057). Female sex (aPR, 0.975; 95% CI, 0.918–1.037; *p* = 0.426), arterial hypertension (aPR, 0.979; 95% CI, 0.923–1.039; *p* = 0.492), and obesity (aPR, 1.009; 95% CI, 0.930–1.096; *p* = 0.821) also did not show statistically significant adjusted associations with resistance (Table 2).

The model converged with all 350 observations. No relevant multicollinearity was detected (maximum VIF, 2.65; mean VIF, 1.72), and the specification test showed no evidence of model misspecification (*p* = 0.381). The deviance and Pearson chi‐square statistics were 50.69 and 26.99, respectively, with 342 residual degrees of freedom. No observation had an absolute standardized Pearson residual greater than 3.

### 3.4. Resistance Profiles

Profile I, comprising resistance to ceftazidime, ciprofloxacin, ceftriaxone, and trimethoprim/sulfamethoxazole, was the most frequent specific combination and occurred in 83 isolates (23.7%). Profile II was identified in 13 isolates (3.7%), while each of the remaining specific profiles involving at least four panel antimicrobials accounted for 2.0% or less of the sample.

Resistance to three profile‐panel agents was recorded in 80 isolates (22.9%), resistance to one agent in 67 (19.1%), and resistance to two agents in 49 (14.0%). Twenty‐seven isolates (7.7%) had no documented resistance to any of the 19 antimicrobials, and eight (2.3%) were resistant only to agents outside the summarized profile panel (Table 3). The distribution of profiles I–XV is shown in Figure 2.

**TABLE 3:** Distribution of antimicrobial resistance profiles among uropathogenic *Escherichia coli* isolates.

Profile	Antimicrobial resistance profile or category	*n*	%
Specific profiles involving ≥ 4 profile‐panel antimicrobials*^a^*			
I	CAZ + CIP + CRO + SXT	83	23.7
II	AMK + CAZ + CIP + CRO + SXT	13	3.7
III	AMK + CAZ + CIP + CRO	7	2.0
IV	CAZ + CIP + CRO + NIT + SXT	5	1.4
V	CAZ + CIP + CRO + NIT	3	0.9
VI	AMK + CAZ + CIP + CRO + CARB	2	0.6
VII	AMK + CAZ + CIP + CRO + CARB + SXT	2	0.6
VIII	CAZ + CIP + CRO + CARB + NIT + SXT	2	0.6
IX	AMK + CAZ + CIP + CRO + NIT	1	0.3
X	CAZ + CIP + CARB + NIT	1	0.3

Other resistance categories*^b^*			
XI	Resistance to three profile‐panel antimicrobials	80	22.9
XII	Resistance to one profile‐panel antimicrobial	67	19.1
XIII	Resistance to two profile‐panel antimicrobials	49	14.0
XIV	No documented resistance to any tested antimicrobial^c^	27	7.7
XV	Resistance only to antimicrobials outside the profile panel^d^	8	2.3

*Note:* Data are *n* (%). Intermediate results were not classified as resistant. AMK, amikacin; CARB, carbapenems; CAZ, ceftazidime; CIP, ciprofloxacin; CRO, ceftriaxone; NIT, nitrofurantoin; SXT, trimethoprim/sulfamethoxazole. CARB denotes resistance to ≥ 1 of ertapenem, imipenem, or meropenem.

^a^Panel comprising AMK, CAZ, CIP, CRO, CARB, NIT, and SXT; profiles involving ≥ 4 agents are shown individually.

^b^Profiles involving 1–3 panel agents were grouped by count.

^c^No resistance to any of the 19 tested agents.

^d^Resistance only to agents outside the panel.

**FIGURE 2:**
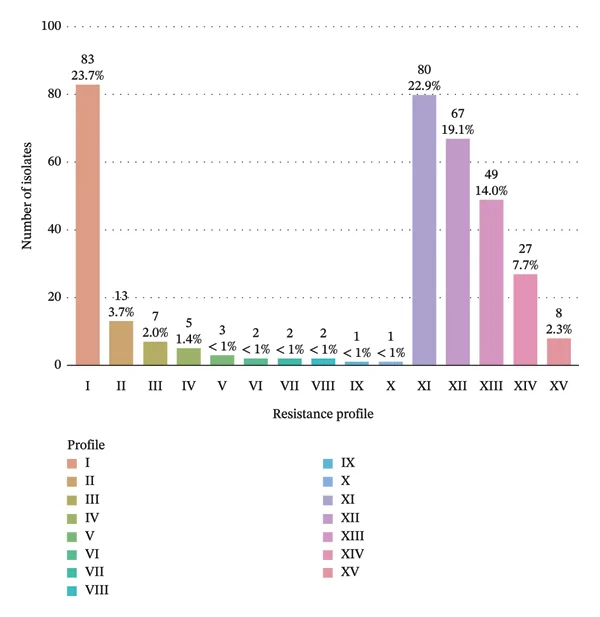
Distribution of antimicrobial resistance profiles among uropathogenic *Escherichia coli* isolates.

Bars represent the number and percentage of isolates in each resistance profile. Roman numerals correspond to the profiles listed in Table 3. Intermediate susceptibility results were not classified as resistant.

## 4. Discussion

This study found that 92.3% of UPEC isolates were resistant to at least one of the 19 antimicrobial agents evaluated. Resistance was highest for ciprofloxacin, ceftriaxone, ceftazidime, and trimethoprim/sulfamethoxazole, whereas amikacin, nitrofurantoin, and carbapenems retained comparatively greater in vitro activity. Age showed a nonlinear association with resistance. After adjustment, no statistically conclusive independent associations were observed for sex, prior antibiotic use, diabetes mellitus, arterial hypertension, or obesity.

The high overall frequency should be interpreted in relation to the broad outcome definition used. This endpoint was intended to capture any documented loss of in vitro activity across the routine panel and is not equivalent to multidrug resistance, extended‐spectrum beta‐lactamase production, or resistance to a specific empirical regimen; it, therefore, encompasses isolates with markedly different susceptibility profiles. Its high prevalence (92.3%), leaving only 27 isolates in the reference group, may have produced a ceiling effect, reducing contrast between outcome groups and limiting the precision of estimates for patient‐level predictors. Accordingly, the composite outcome should not be used alone to select empirical therapy or estimate the burden of more restrictive resistance phenotypes; the drug‐specific susceptibility results and local antibiogram provide the more clinically applicable information. Variability in resistance estimates may also reflect clinical setting, patient selection, prior healthcare exposure, and the antimicrobials included in each laboratory panel [15]. Nevertheless, the 70.9% resistance to ciprofloxacin closely resembles the 70.4% reported among patients with community‐acquired upper UTI at a national hospital in Lima [17].

Comparisons across studies must also consider the methods and interpretive criteria used for antimicrobial susceptibility testing. Automated and minimum inhibitory concentration‐based methods improve standardization, but resistance estimates remain dependent on the tested panel and adopted breakpoints [19]. Differences between CLSI and EUCAST criteria can move isolates between susceptible, intermediate, and resistant categories, with particularly marked discrepancies for selected urinary antimicrobials [20]. The present findings are, therefore, most directly comparable with studies applying similar laboratory standards and outcome definitions.

Peruvian evidence confirms a substantial burden of resistance among UPEC. In Cusco, all isolates selected for extended‐spectrum β‐lactamase production were multidrug resistant, 16.2% met criteria for extensive drug resistance, and no carbapenem resistance was detected [21]. A study involving eight public hospitals found multidrug resistance in 65.7% of the isolates and ESBL production in 55.7%, with high resistance to ciprofloxacin and trimethoprim/sulfamethoxazole [22]. Although these studies evaluated more restrictive resistance phenotypes, their findings are consistent with the frequent coresistance observed in our population.

Geographical heterogeneity is evident even within Latin America. In the Peruvian Amazon, resistance to ciprofloxacin was 37%, whereas resistance to trimethoprim/sulfamethoxazole reached 49% [23]. A 9‐year Colombian study reported ciprofloxacin resistance close to 49% in urinary *E. coli* and documented an increasing temporal trend [24]. These estimates are lower than those found in Piura and support the use of institution‐specific surveillance rather than extrapolation from national or regional averages.

The comparatively low resistance to nitrofurantoin, amikacin, and carbapenems indicates preserved activity against many isolates but does not justify unrestricted empirical use. A Libyan study reported a substantially different susceptibility pattern, illustrating the influence of prescribing practices and local epidemiology [25]. In Mexico, nearly half of urinary *E. coli* isolates produced ESBL, while meropenem retained activity against most strains [26]. These contrasts reinforce the need to interpret susceptibility within the clinical context and local antibiogram.

Evidence from Brazil similarly showed lower resistance to nitrofurantoin, aminoglycosides, and carbapenems. In that setting, hospitalization during the preceding 90 days was the main adjusted predictor of multidrug resistance [27]. Among Peruvian oncology patients with ESBL‐producing *E. coli*, ciprofloxacin resistance exceeded 90%, and blaCTX‐M was detected in most isolates [28]. The selected populations differ from ours, but both studies indicate that healthcare exposure and circulation of resistant lineages may influence the observed phenotype.

The most frequent specific profile in this study combined resistance to ceftazidime, ciprofloxacin, ceftriaxone, and trimethoprim/sulfamethoxazole. In Shanghai, resistance to fluoroquinolones affected 62.3% of UPEC isolates, 39.7% produced ESBL, and no resistance to carbapenems was observed [29]. Long‐term surveillance of more than 30,000 urinary *E. coli* isolates also documented increasing resistance to fluoroquinolones, trimethoprim/sulfamethoxazole, and third‐generation cephalosporins, while nitrofurantoin remained comparatively active [30]. Although molecular testing was unavailable in our study, the concurrent resistance across several classes is compatible with the circulation of isolates carrying multiple resistance determinants.

Patients with resistant isolates were older in the descriptive comparison, but the adjusted association with age was nonlinear. Outpatient data from Washington State showed that the relationship between age and resistance varied by sex and antimicrobial, with stronger associations for ciprofloxacin and ceftriaxone among women older than 50 years [31]. European surveillance likewise identified increasing, decreasing, and nonmonotonic age‐related patterns depending on the organism and antimicrobial evaluated [32]. Thus, the present result should not be interpreted as a constant increase in resistance with each additional year of age.

Older adults may accumulate greater exposure to antibiotics, hospitalization, recurrent infection, urinary instrumentation, and comorbidities, but these factors do not occur uniformly across the life course [33]. At the opposite end of the age spectrum, pediatric surveillance has reported high ESBL production and multidrug resistance in *E. coli*, despite preserved activity of carbapenems and selected urinary agents [34]. Resistant infection is, therefore, not confined to older patients, and age may operate as a marker of differing clinical and exposure patterns rather than as a direct causal determinant.

Diabetes mellitus was associated with resistance in the crude analysis, but the estimate was attenuated after adjustment and its confidence interval included the null value. A systematic review and meta‐analysis found that Type 2 diabetes was associated with greater odds of resistant urinary infection although the included studies differed in population, pathogen, resistance definition, and covariate adjustment [35]. Diabetes may increase susceptibility to complicated or recurrent UTI through glucosuria, impaired immune responses, bladder dysfunction, and more frequent antimicrobial exposure [36]. These mechanisms support biological plausibility but do not establish an independent association in this sample.

A hospital study from Cameroon found that multidrug‐resistant *E. coli* patterns were more frequent among patients with diabetes, particularly when glycemic control was poor [37]. Experimental evidence also indicates that glucosuria can modify UPEC gene expression, enhance biofilm formation, and alter virulence‐related pathways [38]. However, these findings concern susceptibility to infection and bacterial behavior rather than a direct adjusted effect of diabetes on resistance to at least one antimicrobial.

Studies involving patients with diabetes and hypertension have reported a high burden of multidrug‐resistant uropathogens although their adjusted analyses often focused on the occurrence of UTI rather than resistance among infected patients [39]. Other comparisons have found greater drug‐specific resistance among diabetic patients but relied mainly on bivariate analyses [40]. The present estimate should, therefore, be regarded as inconclusive rather than evidence of no possible relationship: its direction was positive, but precision was insufficient to confirm an independent association.

Prior antibiotic use was more common among patients with resistant isolates in the descriptive analysis, yet it was not significant in the adjusted model. A Peruvian case–control study linked prior antibiotic exposure and recurrent UTI with infection caused by ESBL‐producing organisms in older women [41]. Differences may be explained by the broader resistance outcome used here and by limited exposure detail. The database did not consistently record the antimicrobial class, dose, treatment duration, indication, adherence, or interval between treatment and urine culture.

The observed resistance remains clinically relevant despite the absence of multiple independent patient‐level predictors. In a large cohort of patients with *E. coli* bacteremia, resistance to several commonly used antimicrobial classes was associated with higher mortality after adjustment [42]. Urinary sources also represent a substantial proportion of invasive *E. coli* disease, particularly among older and clinically complex patients [43]. These studies do not permit direct inference about outcomes in our population, but they underscore the consequences of delayed or inactive empirical treatment. Prediction studies in elderly patients with sepsis further illustrate the clinical importance of rapidly identifying *E. coli* although their objectives and outcomes differ from antimicrobial resistance surveillance [44].

Several limitations should be considered. The cross‐sectional design precludes causal and temporal inference. The study was conducted at a single referral hospital, and the results may not represent primary care facilities or other Peruvian regions. Prior antibiotic exposure was broadly classified, and information on recurrent UTI, urinary catheterization, previous hospitalization, ICU admission, chronic kidney disease, structural urinary disease, and immunosuppression was not consistently available. The adjusted estimates, therefore, account only for the measured covariates. The absence of statistical significance for sex, prior antibiotic use, diabetes mellitus, arterial hypertension, and obesity should not be interpreted as evidence of no biological or clinical effect. Residual confounding from unavailable factors, together with exposure misclassification and limited precision, may have attenuated or, otherwise, altered the adjusted estimates, and the direction and magnitude of this bias cannot be determined from the available data. This concern is particularly relevant because only 27 isolates had no documented resistance, limiting contrast between outcome groups. The primary outcome did not distinguish isolated resistance from multidrug resistance, and no molecular testing was performed. Strengths include the use of a complete calendar year, random sampling, standardized microbiological records, direct estimation of prevalence ratios, prespecified covariate adjustment, and explicit assessment of the nonlinear age effect. These findings support continued local surveillance, susceptibility‐guided treatment, and multicenter studies integrating detailed exposure histories and molecular characterization.

## 5. Conclusions

UPEC isolates showed a high frequency of resistance, particularly to ciprofloxacin, ceftriaxone, ceftazidime, and trimethoprim/sulfamethoxazole, while resistance to nitrofurantoin, amikacin, and carbapenems remained comparatively low. Age was associated with resistance through a nonlinear pattern, whereas no statistically conclusive independent associations were observed for sex, prior antibiotic use, diabetes mellitus, arterial hypertension, or obesity after adjustment. Given the broad outcome definition and potential residual confounding, these estimates should not be interpreted as excluding biological effects of the measured clinical factors. These findings support the use of local susceptibility data to guide empirical treatment and reinforce the need for continuous antimicrobial resistance surveillance and stewardship.

## Author Contributions

Conceptualization, Christian Alberto Rodriguez‐Saldaña; methodology, Christian Alberto Rodriguez‐Saldaña; formal analysis, Christian Alberto Rodriguez‐Saldaña; investigation, Christian Alberto Rodriguez‐Saldaña, Daniela Valeria Clavijo‐Gallardo, Victor Serna‐Alarcón, Luis Gabriel Farfán‐Chávez, Guiovanna María Wong‐Terrones, and Daniel Reyes‐Chávez; data curation, Christian Alberto Rodriguez‐Saldaña; writing–original draft preparation, Christian Alberto Rodriguez‐Saldaña; writing–review and editing, Daniela Valeria Clavijo‐Gallardo, Victor Serna‐Alarcón, Luis Gabriel Farfán‐Chávez, Guiovanna María Wong‐Terrones, Daniel Reyes‐Chávez; supervision, Christian Alberto Rodriguez‐Saldaña, and Daniel Reyes‐Chávez.

## Funding

This research received no external funding.

## Disclosure

All authors have read and agreed to the published version of the manuscript.

## Ethics Statement

The study was conducted in accordance with the Declaration of Helsinki and approved by the Research Ethics Committee of José Cayetano Heredia III‐1 Hospital, EsSalud Piura (approval code 000016‐CEI‐GR‐RAPI‐ESSALUD‐2025) and by the Institutional Bioethics Committee of Antenor Orrego Private University (Resolution No. 00018‐2025‐UPAO).

## Consent

Informed consent was waived due to the retrospective nature of the study and the use of anonymized electronic medical records.

## Conflicts of Interest

The authors declare no conflicts of interest.

## Data Availability

The data that support the findings of this study are available from the corresponding author upon reasonable request.
